# Infrared needle mapping to assist biopsy procedures and training

**DOI:** 10.1049/htl.2017.0045

**Published:** 2018-01-26

**Authors:** Bruce Shar, John Leis, John Coucher

**Affiliations:** 1 Division of Diagnostic Radiology, Princess Alexandra Hospital, Brisbane, Queensland, Australia; 2 School of Mechanical and Electrical Engineering, University of Southern Queensland, Toowoomba, Queensland, Australia

**Keywords:** infrared imaging, biomedical optical imaging, computerised tomography, infrared needle mapping, biopsy procedures, biopsy training, computed tomography, radiological procedure, CT scan, infrared cameras, cameras, biopsy needle area, needle endpoint, 2D augmentation system, camera pose, multiple low-cost infrared imaging

## Abstract

A computed tomography (CT) biopsy is a radiological procedure which involves using a needle to withdraw tissue or a fluid specimen from a lesion of interest inside a patient's body. The needle is progressively advanced into the patient's body, guided by the most recent CT scan. CT guided biopsies invariably expose patients to high dosages of radiation, due to the number of scans required whilst the needle is advanced. This study details the design of a novel method to aid biopsy procedures using infrared cameras. Two cameras are used to image the biopsy needle area, from which the proposed algorithm computes an estimate of the needle endpoint, which is projected onto the CT image space. This estimated position may be used to guide the needle between scans, and results in a reduction in the number of CT scans that need to be performed during the biopsy procedure. The authors formulate a 2D augmentation system which compensates for camera pose, and show that multiple low-cost infrared imaging devices provide a promising approach.

## 1 Introduction

Biopsies are a useful and commonly used tool in medicine, whereby a tissue sample is taken in order to be externally analysed to test for diseases such as cancer. Biopsies on tissue near the surface of the body can be easily performed; however, when the tissue is deep inside the body, the biopsy becomes much more difficult. In these cases, a computed tomography (CT)-guided biopsy may be undertaken. A CT scan provides a cross-sectional view of the body, with a series of these images taken in what is known as a CT study, to visualise the location of both the suspect tissue and the needle being used to extract the biopsy sample. During a biopsy, the needle is placed in the patient's body and all necessary adjustments are done to align the needle perfectly with the predicted target prior to piercing the skin. The anticipated path of the needle is traced on a series of CT scans extrapolating the needle towards the lesion.

## 2 Problem formulation

As a result of the number of repeated scans required to guide the needle down the correct path towards the lesion of interest, reducing the radiation dose arising from multiple CT scans has been identified as an important goal [1]. Furthermore, there is a clear demand for a natural and intuitive system which could play a major role in the guidance of surgical procedures [2]. During the CT-guided biopsy procedure, multiple CT scans are taken to accurately identify the needle trajectory; these result in radiation exposure to the patient and staff [3]. There is evidence that radiation doses from commonly performed diagnostic CT examinations are higher than generally assumed [4]. Furthermore, the radiation exposure resulting from CT examinations is highly variable, with up to an order of magnitude difference reported across physicians [5]. Thus, CT examinations increase the lifetime risks associated with radiation exposure, with younger patients at higher risk.

As noted in [6], guidance devices for interventional radiology represent an area of great interest, with some systems emerging to address this need. Although different approaches are possible, our initial system which was to project a full 3D image was found to be less acceptable to practitioners than one involving a 2D overlay of the conventional CT display. This paper proposes a low-cost measurement technique which can estimate the location of a biopsy needle endpoint between successive CT scans. This involves a training procedure in which the mapping from images of the needle to the corresponding location in the DICOM image [7] is determined. Once this mapping is known the needle endpoint can be predicted using only images of the needle. The goal is to reduce the cumulative radiation exposure to patients undergoing such procedures. The specific emphasis is on the design of a low-cost system which can be immediately employed as an adjunct to existing systems.

## 3 Research context

Although image-guided surgery has received a great deal of attention in the last two decades and has led to practical systems becoming widely used, guidance and imaging for biopsy procedures has arguably received less attention. Biopsy guidance using images has recently been reported [8], but in the main, biopsy guidance has been limited to conventional ultrasound. Since a common modality for biopsy feedback is ultrasound, it is logical to attempt to locate the biopsy needle using the same modality, though the inherent physical nature of ultrasound presents a different localisation problem [9].

Fusion of optical and ultrasound imaging for the purposes of biopsy needle guidance was attempted in [10], using a precomputed mapping of 2D images to the measured needle position in 3D. A recent study addresses planning of needle access pathways with respect to usability and target accessibility, noting that visualisation of the planned access pathways has drawn little attention [11].

The feasibility of CT-guided bone biopsies using a novel robotic needle guide was evaluated in [12]. Laser navigation systems are another possibility, as reported in [13]. Many current navigation systems are electromagnetic, not optical or infrared. Such systems do not have a line of sight limitation [14]. Electromagnetic trackers use a field generator along with a number of small coils embedded in surgical instruments [15]. The shortfall of electromagnetic trackers is that the surgical environment must be devoid of any ferromagnetic material that could interfere with the electromagnetic field and degrade the measurement accuracy. Another problem with electromagnetic trackers [16] is that some of the surgical instruments need to be modified to include sensor coils.

Recently, an economic evaluation of the benefits of using a commercial guidance system for the specific case of lung biopsies was reported in [17], which noted the very substantial costs of such a system and the difficulty in justifying these. A payback time of the order of 4 years was estimated. Finally, requirements for neurosurgery CT-guided procedures are reported in [18], with accuracy of the order of 5 mm reported.

## 4 Scope and aims

The proposed system does not use stereotactic equipment and braces, nor does it have a constant feed from the ultrasound scanner. It provides guidance to the radiologists while they are advancing the needle towards the lesion of interest. It employs non-harmful infrared imaging, and promises to be able to address the goal of exposure reduction. A prototype system has been designed and tested, and we present initial results here using a two-camera system. As a result of the testing performed, avenues for further work to enhance the accuracy of the system are suggested. The primary goals were firstly, to determine whether such relatively low-cost and medium-resolution IR cameras could provide sufficient accuracy for CT training and possibly even actual CT procedures, and secondly to determine, in consultation with radiologists, the best way to usefully display such procedural augmentations.

## 5 Current methods and limitations

**Fig. 1:**
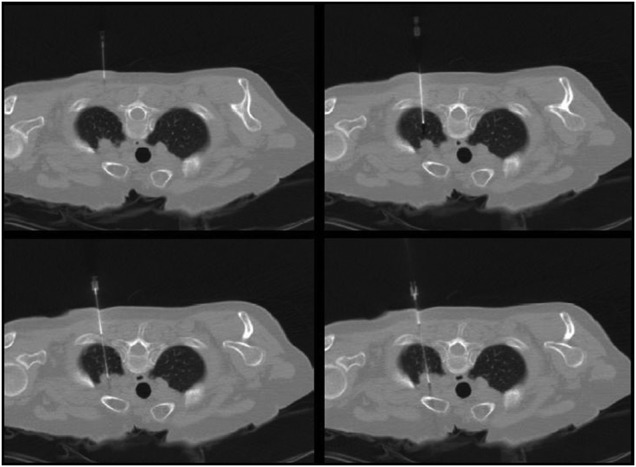
Cross-sectional image of the body with the biopsy needle being progressively inserted. Successive CT images provide visualisation of the needle location, and as such provide critical guidance to the specialist

**Fig. 2:**
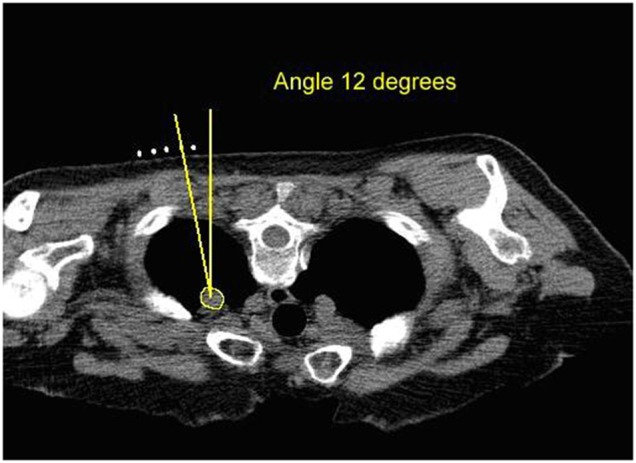
Planned biopsy path showing the desired entry angle

Current techniques rely on multiple CT scans to accurately place a biopsy needle as shown in Fig. 1. One of the disadvantages of the existing freehand biopsy procedure is the problem of accurately placing a biopsy needle at the required angle. This is mainly due to the lack of spatial referencing for the operator and the weight of the needle, which can impact the angle of the biopsy needle during a CT scan. A CT guided biopsy procedure involves the following steps [19]:

- Performing the CT scan by first positioning the patient and applying skin markers.
- Identifying a safe biopsy path by assessing the CT images and calculating the entry angle and identifying the skin entry point (Fig. 2).
- Identifying the biopsy entry point on the patient.
- Using an estimated angle inserting the biopsy needle.
- Progressing the needle in a stepwise fashion, taking more CT images from the patient at each step to identify any needed corrections in trajectory.
- Confirming the position of the needle prior to taking a sample of the lesion of interest.

There are also some biopsy scenarios where radiologists use a ‘double angle’ method to conduct the biopsy. Double angled biopsy is when the lesion being biopsied is difficult to access safely and as such the needle is angled in two planes, left/right and up/down. In order to get to the lesion of interest, very precise angles are needed. Currently, the CT gantry is tilted to the required angle. The clinician then advances the needle into the patient, and uses the CT lasers as a guide for the up/down angle as required. However, newer CT scanners are unable to tilt their gantry because the CT scanners have been increasing in size. Thus, there is a need for an alternative process of conducting the double angled biopsies under CT guidance. Our proposed method does not suffer from these limitations.

## 6 Equipment and setup

**Fig. 3:**
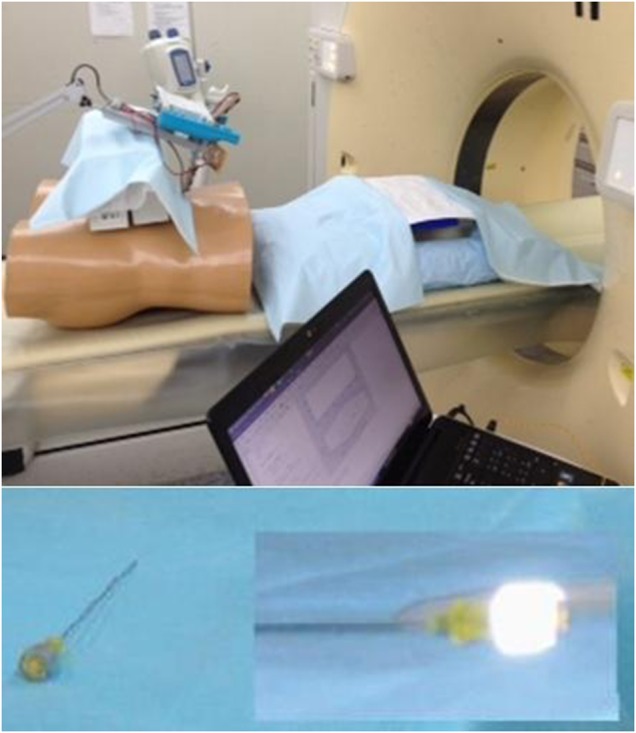
Experimental setup. The CT scanner is on the right, and the IR cameras to the left

In a major metropolitan hospital, a medical phantom was set up inside a medical imaging CT scanner with the infrared cameras fixed at an appropriate angle as shown in Fig. 3. The setup was orchestrated in a way to mimic a real biopsy procedure that regularly takes place in that environment using the same CT scanner. The operators assisting in these experiments were trained radiologists who perform biopsies on a regular basis. The operator was positioned to the left or the right of the phantom, much like a real biopsy scenario.

**Fig. 4:**
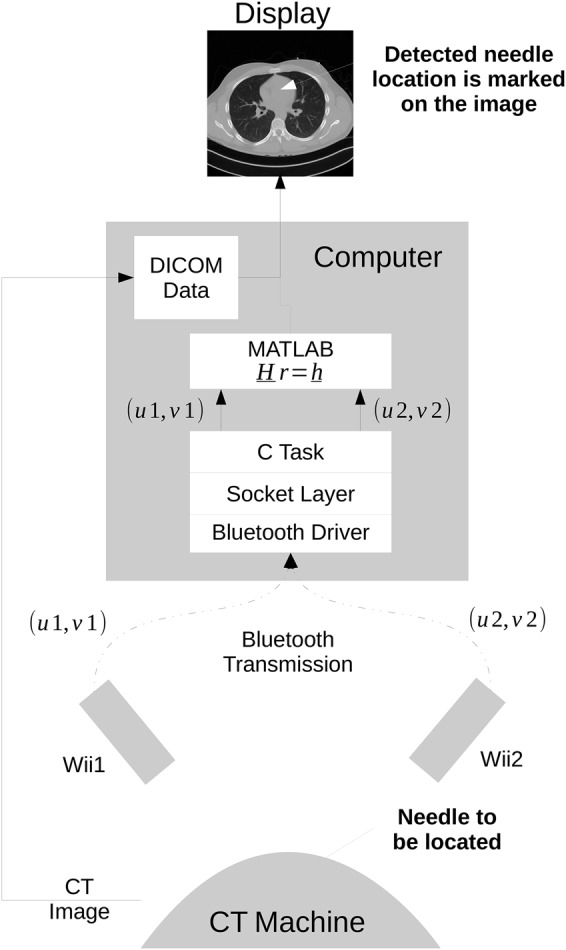
Data flow and computation required in the proposed infrared guidance system. The CT image (top) is the reference. Communication from the IR cameras is done via Bluetooth. Computational aspects required for the needle image estimation are currently performed using MATLAB

The Nintendo Wii Remote infrared sensor was chosen due to availability, cost effectiveness, ease of interfacing, and resolution. The infrared sensor on the Wii Remote is able to track up to four IR hotspots simultaneously, with positions output at 100 Hz with $1024 \times 768$ interpolated resolution [20]. Only the positions and size/intensity of the IR hotspots are output, with the raw video output from the sensor not available. A Bluetooth interface is included on the Wii Remote to enable communication as depicted in Fig. 4. This communication is performed using TCP/IP sockets. The data captured from the device driver is consumed by a task which interfaces with ${\rm MATLA}{\rm B}$^®^ for the numerical processing and graphical display.

## 7 Algorithms and processing

The initial approach was to provide a full three-dimensional augmentation to the biopsy procedure. This system worked well in terms of algorithmic evaluation, but it was discovered that radiologists preferred a 2D augmentation of existing systems. To this end, algorithms to effectively produce the display using multiple-camera approach had to be designed. One possible approach is that of considering a least-squares model, as shown in [21]. The sparseness of the representation made this approach infeasible.

The most general version of the problem requires estimating the camera calibration parameters (focal length, principal point coordinates, aspect ratio, and skew). This can be established with a set of correspondences using the well-known direct linear transform (DLT) algorithm [22]. There is an extensive list of different algorithms that improve the accuracy of the DLT. The generic family of methods to solve this *n* 3D point to 2D point calibration problem when the intrinsic camera calibration parameters are known as perspective-*n*-point methods. The accurate determination of camera parameters, either known or by calibration methods, is undesirable in a clinical setting, where the additional setup time cannot be justified. Thus, fully automatic means are required. The determination of camera pose and focal length estimation is not a new problem in computer vision, with *n* 3D-to-2D point correspondences utilised [23].

The proposed method does not require the camera intrinsic or extrinsic parameters [24]. Initial efforts focussed on reconstructing a 3D view of the needle within the body. However, after feedback from radiologists it became clear that for guidance purposes, a 2D projection was potentially of more practical use.

The approach taken is based on the calibration approach of [25] as described in [26]. Although many other approaches to the problem have been examined (such as [27]), the solution of [25] is elegant and does not require iterative refinement nor gradient information.

**Fig. 5:**
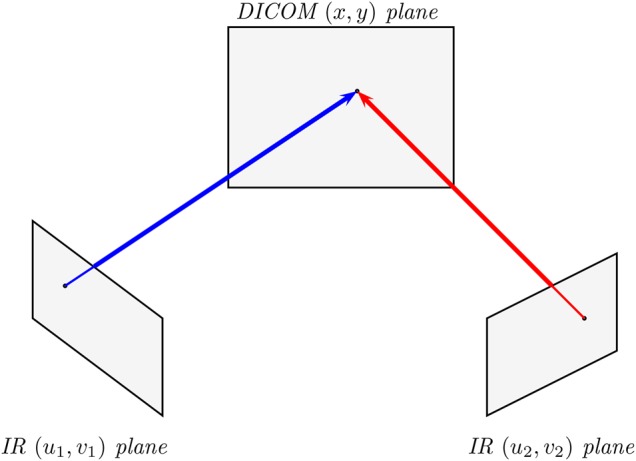
Mapping the IR camera planes (u, v) to the DICOM image point (x, y)

The basic problem may be summarised by referring to Fig. 5. The projection from a single camera imaging plane onto a DICOM image is shown, and this is extended to two cameras as described below.

Point ${\bi m}_i = \lsqb{u_i^{\lpar k\rpar } v_i^{\lpar k\rpar } } \rsqb^{\rm T}$ reported from the IR camera(s) *k* are mapped onto the DICOM plane $x_i$, $y_i$ using

(1) $$s\left({\matrix{ {u_i} \cr {v_i} \cr 1 \cr } } \right)= \left({\matrix{ {\,p_{11}} & {\,p_{12}} & {\,p_{13}} \cr {\,p_{21}} & {\,p_{22}} & {\,p_{23}} \cr {\,p_{31}} & {\,p_{32}} & {\,p_{33}} \cr } } \right)\left({\matrix{ {x_i} \cr {y_i} \cr 1 \cr } } \right)\eqno\lpar 1\rpar $$

This is assumed to be a linear translation, rotation and scaling defined by

(2) $$s\widetilde{{\bi m}} = {\bi P}\widetilde{{\bi M}}\eqno\lpar 2\rpar $$

where *s* is an (arbitrary) scaling factor and ${\bi P}$ is the $3 \times 3$ projection matrix. Writing equations in terms of components in a matrix form, we have

$$\matrix{ {\left({\matrix{ {x_i} & {y_i} & 1 & 0 & 0 & 0 & { - x_iu_i} & { - y_iu_i} & { - u_i} \cr 0 & 0 & 0 & {x_i} & {y_i} & 1 & { - x_iv_i} & { - y_iv_i} & { - v_i} \cr } } \right)} \cr } \left({\matrix{ {\,p_{11}} \cr {\,p_{12}} \cr {\,p_{13}} \cr {\,p_{21}} \cr {\,p_{22}} \cr {\,p_{23}} \cr {\,p_{31}} \cr {\,p_{32}} \cr {\,p_{33}} \cr } } \right)= \left({\matrix{ 0 \cr 0 \cr } } \right)$$

This single-camera matrix equation is thus ${\bi G} {\bi p} = {\bf 0}$, where ${\bi G}$ is the $2 \times 9$ matrix of known parameters, ${\bi p}$ is the $9 \times 1$ vector of coefficients of the ${\bi P}$ calibration matrix, and ${\bf 0}$ is the $2 \times 1$ zero vector.

Next, for a set of observations, we stack all ${\bi G}_i$ sets together to yield

(3) $$\left({\matrix{ { - - - - - - } & {{\bi G}_1} & { - - - - - - } \cr { - - - - - - } & {{\bi G}_1} & { - - - - - - } \cr {} & \vdots & {} \cr { - - - - - - } & {{\bi G}_n} & { - - - - - - } \cr } } \right)\left({\matrix{ \vert \cr \vert \cr {\,p_{ij}} \cr \vert \cr } } \right)= \left({\matrix{ 0 \cr \vdots \cr 0 \cr } } \right)\eqno\lpar 3\rpar $$

Solving $\min _p\vert \vert {\bi Gp}\vert \vert _2$ corresponds to finding the minimum eigenvalue $\lambda $ of ${\bi G}^{\rm T}{\bi G}$, and the solution is the corresponding eigenvector. Thus here we have ${\bi G}^{\rm T}{\bi G}$, and the corresponding eigenvector is the estimate for ${\bi p}$, which is then reshaped into the matrix ${\bi P}$.

Once ${\bi P}$ has been determined, we can map a given (*u*, *v*) into (*x*, *y*). There will, however, be error in any measurements we take and this will result in errors in the estimated positions. We can reduce the error by using multiple cameras, then finding the estimated position which best matches all observations.

Now denote $p_{ij}^{\lpar k\rpar } $ as parameter $p_{ij}$ for camera *k*. Thus for two cameras

(4) $$\left({\matrix{ {\,p_{11}^{\lpar 1\rpar } } & {\,p_{12}^{\lpar 1\rpar } } & { - u^{\lpar 1\rpar }} & 0 \cr {\,p_{21}^{\lpar 1\rpar } } & {\,p_{22}^{\lpar 1\rpar } } & { - v^{\lpar 1\rpar }} & 0 \cr {\,p_{31}^{\lpar 1\rpar } } & {\,p_{32}^{\lpar 1\rpar } } & { - 1} & 0 \cr { - - } & { - - } & { - - } & { - - } \cr {\,p_{11}^{\lpar 2\rpar } } & {\,p_{12}^{\lpar 2\rpar } } & 0 & { - u^{\lpar 2\rpar }} \cr {\,p_{21}^{\lpar 2\rpar } } & {\,p_{22}^{\lpar 2\rpar } } & 0 & { - v^{\lpar 2\rpar }} \cr {\,p_{31}^{\lpar 2\rpar } } & {\,p_{32}^{\lpar 2\rpar } } & 0 & { - 1} \cr } } \right)\left({\matrix{ x \cr y \cr {s^{\lpar 1\rpar }} \cr {s^{\lpar 2\rpar }} \cr } } \right)= \left({\matrix{ { - p_{13}^{\lpar 1\rpar } } \cr { - p_{23}^{\lpar 1\rpar } } \cr { - p_{33}^{\lpar 1\rpar } } \cr { - - } \cr { - p_{13}^{\lpar 2\rpar } } \cr { - p_{23}^{\lpar 2\rpar } } \cr { - p_{33}^{\lpar 2\rpar } } \cr } } \right)\eqno\lpar 4\rpar $$

which is of the form

(5) $${\bi Hr} = {\bi h}\eqno\lpar 5\rpar $$

Since ${\bi H}$ is not square, it cannot be inverted in the conventional sense to find ${\bi r}$. In most cases there will be no value for ${\bi r}$ so that the equation is exactly true, so a value of ${\bi r}$ is chosen that makes the equation closest to being true. In this case, ${\bi H}$ is an overdetermined matrix, and the aim is to compute ${\bi r}$ so that the squared error between ${\bi Hr}$ and ${\bi h}$ is minimised. We wish to find vector ${\bi r}^ \ast = \min _{\bi r}\vert \vert {\bi Hr} - {\bi h}\vert \vert _2$.

Such a system may be solved using various approaches, either directly or iteratively. A problem with iterative solutions is usually the need to compute gradients, which can be problematic with sparse and/or noisy data. For the present work, we have employed a least-squares formulation (the Moore-Penrose pseudoinverse) which provides a direct solution

(6) $${\bi r}^ \ast = \lpar {\bi H}^{\rm T}{\bi H}\rpar ^{ - 1}{\bi H}^{\rm T}{\bi h}\eqno\lpar 6\rpar $$

The vector ${\bi r}^ \ast $ provides the scaled location of the needle endpoint on the DICOM image.

## 8 Results

Many authors have noted the difficulty in comparing and evaluating the concept of accuracy within medical image domains. The authors in [28] discuss the issues extensively, in the context of competing commercial systems. We note that the results presented here are preliminary for the low-cost system described, and that the potential is clearly demonstrated, especially as a training aid for radiologists.

A set of experiments was designed to show that unseen points can be estimated with sufficient accuracy, as well as to quantify the error between the actual location of a point and the location predicted by the algorithm. To test the model, two infrared cameras were set up to record the locations of a needle tip. The location of the needle tip is also measured using X-ray imaging and the position read from the DICOM image. The DICOM image provides the 2D target location (*x*, *y*), while the pixel locations measured from the infrared camera provides the training data. In these experiments, a target was placed inside a phantom at a random spot. The radiologist performing the experiment took a series of CT scans to identify the target, which was $ \simeq 100\; {\rm mm}$ inside the phantom. This depth was chosen to mimic a typical lung biopsy. Using the software and display provided, the needle was advanced towards the target. A second series of CT scans confirmed the distance from target, with the distance measured precisely. The process was repeated from different entry points and sides.

**Fig. 6:**
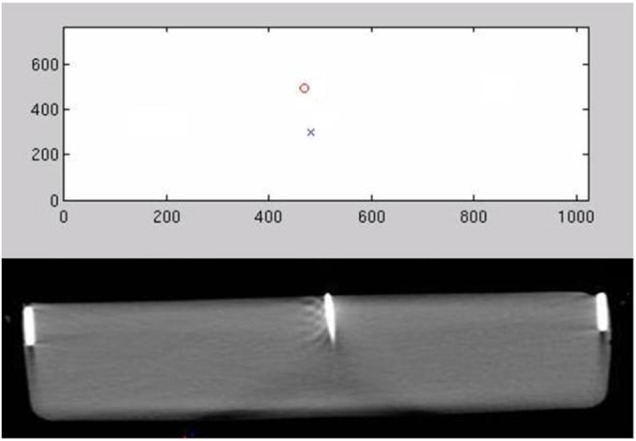
Location of the needle tip identified using two infrared cameras, with the true DICOM CT-scanned image below. In the top image, the x and o are live outputs from the IR cameras, which demonstrate the location of the needle using the reflection from two infrared reflective tapes. The dimensions of the top image are the raw locations from the IR camera, which are converted to an image location using the method proposed in this paper

**Fig. 7:**
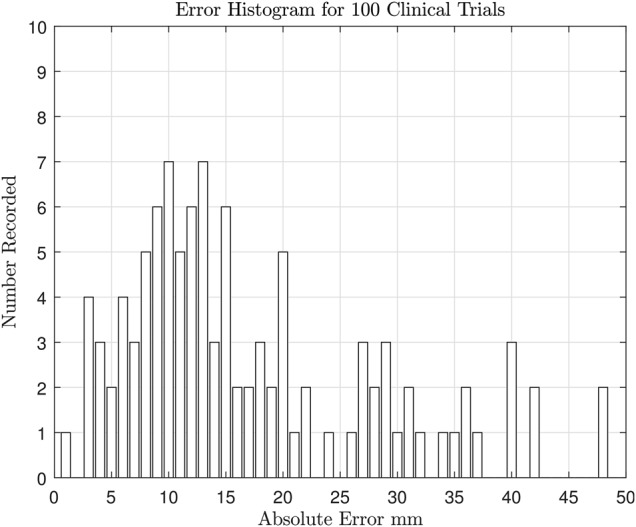
Error histogram showing the error from the experimental trials with a full insertion depth of $ \simeq 100\; {\rm mm}$. The error is defined as the position estimated by the radiologist using the proposed assistive image display as compared to the precise position obtained using a CT scan. This mode of operation may be suitable for training purposes, with an error of the order of 1–3 mm over the expected inter-scan depth of 10 mm

An example showing both the estimated position and the true position is shown in Fig. 6. This evaluation over a full biopsy distance is for ascertaining the usefulness of the approach and to gauge the accuracy, though it does not mimic the guidance method envisaged for actual biopsies. This is because the anticipated needle insertion between scans, with one scan omitted and using IR guidance only, is of the order of 10 mm. As shown in the results of Fig. 7, the expected error in this scenario is of the 13 mm, or 1.3 mm over the stated insertion range. Thus we anticipate that accuracies of the order of 1–3 mm could be achieved in practice.

To measure the effectiveness of this approach, we use leave one out cross-validation. This involves using 99 points to train the model, then testing it on the 100th point. The predicted location can then be compared to the actual measured location. This is repeated for every sample (100 times). By doing it this way it can be ensured that no training information is ever used for testing, as this would give unrealistically good results.

This histogram shows all but four outliers, and the estimation using IR guidance alone is generally good. We attribute the outliers to operator unfamiliarity in using the new positioning prototype, as well as unexpected reflection from extraneous objects.

## 9 Discussion

The results presented for the full insertion depth show considerable promise for such a medium-resolution camera approach. It must be noted that several enhancements to the software and presentation mode would be required for clinical usage. First, quantification of the precise errors between successive scans for short insertion depths needs to be evaluated. For the results presented over the full insertion depth, the relative error was considered quite good. The error bounds would also need to be evaluated and displayed graphically. The use of more than two IR cameras is also a matter of further investigation. The current setup employs two cameras; however, more than two can easily be accommodated by the algorithm. Better solution of the projection equations may also lead to improved results. The closed-form solution for both calibration and estimation works satisfactorily; however, further refinements to these methods are given in the literature. Specifically, iterative refinement may further reduce the numerical inaccuracies.

## 10 Conclusions

This paper described the problem of radiation dosage in CT-assisted biopsy, and a possible solution was presented. This uses an infrared camera pair, an infrared reflector attached to the biopsy needle, and an algorithm to project the estimated needle position onto the CT image. This extended algorithm computed a scaled estimate of the needle endpoint and assists with navigating the biopsy needle through a dedicated and custom built graphical user interface. A series of experiments similar to real-life biopsy scenarios were conducted using a phantom, biopsy needle and CT scanner and results were evaluated. Clinical evaluation for training purposes was also investigated, with positive feedback provided by radiologists. The approach presented is extensible to more than two cameras, so as to attain greater accuracy and also to address the issue of possible occlusion of the cameras.

## 11 Funding and declaration of interests

None declared.
